# Incidence of endosymbiont bacteria *Wolbachia* in cowpea weevil *Callosobruchus maculatus* Fabricius (Coleoptera, Chrysomelidae)

**DOI:** 10.1371/journal.pone.0313449

**Published:** 2024-12-10

**Authors:** Bilal Rasool, Tahira Younis, Saba Zafar, Aqsa Parvaiz, Zeeshan Javed, Inshaal Rasool, Muhammad Shakeel

**Affiliations:** 1 Department of Zoology, Faculty of Life Sciences, Government College University Faisalabad, Punjab, Pakistan; 2 Department of Biochemistry and Biotechnology, The Women University Multan, Punjab, Pakistan; 3 Department of Bioinformatics, Faculty of Life Sciences, Government College University Faisalabad, Punjab, Pakistan; 4 Pakistan Agricultural Research Council, Islamabad, Pakistan; Bayero University Kano, NIGERIA

## Abstract

This study focuses on the cowpea weevil, *Callosobruchus maculatus*, a globally distributed grain pest that affects cereals and pulses. Using chemicals to store grains can harm pest control and pose risks to consumers and the environment. The facultative intracellular symbiont bacteria *Wolbachia* can affect host’s reproductive capacities in a variety of ways, which makes it useful in the management of pests such as *C*. *maculatus*. The main goal of the study was to identify *Wolbachia* diversity in the *C*. *maculatus* population. Phylogenetic analysis utilized mitochondrial *COI* and *12S rRNA* genes to identify the host *C*. *maculatus*, while screening for *Wolbachia* was conducted using genes (*wsp*, *coxA*, and *ftsZ*) genes. Molecular phylogenetic analysis of the *Wolbachia* genes resulted in one new *Wolbachia* strain (*w*Cmac1) in *C*. *maculatus* populations and contrasting already published data of other *Callosobruchus* strains. The study discussed the detection of *Wolbachia* and its phylogenetic comparison with other *C*. *maculatus* and Coleopteran populations. It is important to take these findings into account when considering host-pathogen interactions.

## 1. Introduction

Pulses are more affordable source of protein for many people around the world, particularly in developing countries [1]. Because some varieties contain even more protein, they are significant and essential in terms of nutrition [2]. Pulses are recognized for their effective and multiple participatory roles in enhancing environmental and agricultural sustainability [1, 3].

The potential pest control quality, quantity, and actual benefits of pulse pests for meeting global and national targets are essential to supporting policy development and integrated pest control planning. Priorities for global strategic research on pulse crops include end*-*user needs, transformative potential, and sustainability. To effectively control pests, research is required to develop a clear understanding of pest management, environmentally friendly solutions, and socioeconomic benefits [4].

Insects comprised above seventy percent of species of arthropods and Coleopterans [5]. According to estimation, about 70 to 95% of all beetle species remain undescribed [6]. Beetles are the model organisms often used in biological, medical, and environmental research [7]. Among the stored grain pests, the bruchids are essential species in pulses during storage [8]. Cowpea weevil *Callosobruchus maculatus* F. (Coleoptera: Chrysomelidae: Bruchinae) has worldwide distribution except for Antarctica. This agricultural pest ranges throughout the tropical and subtropical world [9–11].

*Callosobruchus maculatus* is one of the important factors for economic loss to the pulses and a substantial pest of stored grains [8, 12–14]. It is a model organism in population biology and affects all types of pulse fields. This pest often originates in pulse debris, may build up huge populations, and has emerged as the principal post*-*harvest pest. Moreover, this pest multiplies rapidly within a short time and can cause quantitative and qualitative losses of up to 100% of stored pulses [8, 14–16].

The utilization of synthetic chemicals is a common practice to control pests on pulses during storage [17]. These strategies led to several health risks, such as residual toxicity, ecological contamination, natural imbalances, pollution of the environment, insect resistance, secondary insect epidemics, phytotoxicity, chemical residues in food, and disruption of biological fauna [15, 17–19]. Consequently, there is a need to integrate advanced biotechnological tools to control this pest.

Endosymbiotic microorganism associations are quite pervasive in insects and arthropods [20]. There is diversified interdependency present during the interactions of the host and endosymbiotic relationships [21]. *Wolbachia* alpha*-*proteobacteria, detected in many species of arthropods, can transmit maternally. *Wolbachia* has been detected in 10–70% of examined hosts [21–22]. *Wolbachia* in Coleopteran species is well established and documented [23].

It has gotten attention due to its vast occurrence and possible applications in pest and disease management. *Wolbachia* is well known for causing reproductive changes in host tissues through mechanisms such as parthenogenesis, feminization, male killing, and cytoplasmic incompatibility (CI) [24]. Cytoplasmic incompatibility (CI) occurs when a bacterium causes sterility in crosses between *Wolbachia-*infected males and wild females. This can be used to control insect pests through the incompatible insect technique (IIT). Releasing only males is crucial for a successful IIT*-*based approach. Previous research has shown positive effects of IIT on insect disease vectors and agricultural pests. *Wolbachia-*based IIT could be an effective and environmentally friendly pest management approach [25–28]. Consequently, *Wolbachia* is regarded as a selfish genetic element for its engagement with the host in an evolutionary sense [24], making it an attractive subject of evolutionary and molecular biology [24, 29]. Therefore, if the endosymbiotic microorganism prevails in *C*. *maculatus*, it will offer us a good configuration to explore the host*-*symbiont dynamics [23, 30].

For population genetic and phylogenetic research in a variety of organisms, insect mtDNA, which is inherited maternally, is an invaluable resource [31–33]. The advent of polymerase chain reaction PCR has made primers for mitochondrial genes amplification accessible [32, 34]. Mitochondrial assessment for the characterization and identification of different arthropod species is frequently used. For insects and related groups, *12S rRNA* fragments are universal. For the identification of insect species, the cytochrome c oxidase subunit I (*COI*) DNA barcode region is thought to be a useful and reliable diagnostic tool because of its fast mutation rate to differentiate closely related species [35]. *Wolbachia* outer surface protein (*wsp*) is considered to be the valid markers for the exploration of endosymbiotic bacteria [36]. In comparision, multilocus strain typing (MLST) is an appropriate and fast detectable approach that is recognized for exploring bacterial strains, including *Wolbachia*. MLST exploits five conserved housekeeping genes, including *coxA*, *gatB*, *ftsZ*, *hcpA* and *fbpA* [37, 38]. This approach is a promising tactic for exploring and cataloging strains and studying the molecular ecology, biodiversity, and development of *Wolbachia* [39–41]. There are currently 17 *Wolbachia* supergroups known to exist and identified as A to R [24, 42, 43]. Focused research objectives on *Wolbachia* had great potential for possible application in agricultural pest management and vector borne diseases. The application of chemical control used for the control of *C*. *maculatus* [44, 45] jeopardized the resistant populations, human health, and the environment [45]. There were already numerous insect induced pest incursions of stored grains in the storage area. Controlling stored product insects primarily aims to eradicate these residual populations. Ecologically risky methods of controlling bruchids are not very suitable for small*-*scale, economically viable farmers [46]. *Wolbachia* manipulates its host’s biology through phenotypic alterations, including cytoplasmic incompatibility [24]. This leads to the incompatible insect technique (IIT), which effectively controls insect disease vectors and agricultural pests [25, 26, 28]. The current study set out to look into *Wolbachia* infection in the various natural populations of *C*. *maculatus*. Nevetheless, molecular identification of *C*. *maculatus* through mitochondrial (*COI*) and *12S rRNA* genes was accomplished. Phylogenetic analysis was performed using marker gene sequences. *Wolbachia* strains were screened to test the hypothesis that advanced typing (*wsp*, *coxA*, and *ftsZ*) genes in addition to (*12S rRNA*, *COI*) of the *C*. *maculatus* populations. This investigation represents the first step toward a longer*-*term objective of determining whether *Wolbachia-*induced strains can be used as a novel, eco*-*friendly tool for *C*. *maculatus* management.

## 2. Materials and methods

The present research was conducted to explore the endosymbiont bacteria *Wolbachia* in *Callosobruchus maculatus* populations collected from different regions (Faisalabad, Lahore, Multan, Peshawar, and Hyderabad) of Pakistan and its phylogenetic narrations. Geographical locations of the collection sites can be accessed from https://www.esri.com/en-us/arcgis. The research experiments were conducted in the Department of Zoology, Government College University, Faisalabad, Punjab, Pakistan. The samples were collected from grain storage facilities and preserved in 96% ethyl alcohol at 4°C before the molecular experiments. Rearing was also conducted under controlled laboratory conditions at 25 ± 2°C and 70 ± 5% RH. Molecular experiments were steered by using mitochondrial and three *Wolbachia* including two MLST genes to investigate the population dynamics of *C*. *maculatus* and *Wolbachia* infection status in five different localities in Pakistan. The cowpea weevil (*C*. *maculatus*) was identified using molecular DNA barcoding techniques and morphological characteristics under a microscope.

### 2.1. DNA extraction

Twenty two to twenty four individuals from each locality were utilized for DNA extractions through extraction kits (Sangon Biotech, China) by following the protocols provided by the product company. DNA quantification was performed by the nanodrop method. The DNA of the samples was precipitated with ethanol for purification purposes following the standardized protocol.

### 2.2. Polymerase Chain Reaction (PCR)

Samples were amplified through the *12S rRNA* gene with primer sets (S2 Table). The samples of *C*. *maculatus* populations were passed through PCR tests for barcoding and phylogenetic analysis with the cytochrome oxidase I gene in a mixture of 25 μL with both forward and reverse sets of primers (S2 Table). The genes characterization was performed in 25 μL volume reactions containing 2.5 μL of 10× PCR buffer (Fermentas), 2.0 μL MgCl_2_ (2.5 mM), 0.2 μL dNTPs (200 μM), 1 μL of Taq Polymerase (1U/μL), 1 μL of each primer, 1 μL of extracted DNA, and 16.3 μL of dsH_2_O. PCR was conducted for 2 min at 95°C to characterize the *COI* mitochondria. Thereafter, under a temperature profile of 95°C for 10 min followed by 30 cycles of 95°C for 30 s, 60°C for 30 s, and 72°C for 1 min and a final extension lasting 15 min at 68°C. The *12S rRNA* gene fragment amplification was carried out at initial denaturation at 94°C for 15 min, followed by 45 cycles of denaturation at 94°C for 45 s, annealing at 42°C for 45 s, extension at 72°C for 45 s, and a final extension at 72°C for 10 min. During the amplification of the *coxA* (MLST) gene, the PCR process started at 95°C for 2 min, then it went through 32 cycles of 30 s at 94°C, 45 s at 55°C, 1 min at 72°C, and a final extension lasting 15 min at 68°C. The temperatures (T_m_) for the *ftsZ* (53°C) and *wsp* (55°C) genes were maintained (S2 Table). In horizontal gel documentation, DNA fragments were used with a 1X TAE running buffer for the bands’ image pattern. Gels with 1–2% agarose and 3–4 μL ethidium bromide were utilized. DNA bands were observed on a UV transilluminator. PCR conditions, primer details, T_m_ information, obtained products of all tested five genes (*COI*, *12S rRNA*, *wsp*, *coxA*, and *ftsZ*) are mentioned in the supplementary (S2 Table). Due to products of variable size and low titer *Wolbachia* density the PCR products of *wsp* gene from one representative sample of each population were cloned following the procedures of Arthofer et al. [47]. Plasmid DNA was purified using a Qiagen kit, and Sanger sequencing was performed using the ABI 3730 DNA analyzer (Applied Biosciences, Foster City, CA, USA). The obtained plasmids were manually edited, aligned using ClustalW [48] and compared with *Wolbachia* sequences from GenBank using BLAST analysis.

### 2.3. Phylogenetic sequence data analysis

The retrieved sequences from the present study were compared with different other sequences from Genbank data of *C*. *maculatus* within the same gene and from different countries. *Wolbachia* sequences from (*wsp*, *coxA*, and *ftsZ*) genes were compared with GenBank data of the same genes from different origins and *Wolbachia* strains to identify *Wolbachia* supergroup and strain diversity. Nucleotide sequences from the gene bank NCBI database were aligned and compared for phylogenetic tree analysis through ClustalW version 2.0.9 [48–50]. The tree topologies were analyzed by using the Neighbor*-*joining (NJ) implementation with the Tamura*-*Nei genetic distance model of Geneious software 2024 R11 version (https://www.geneious.com) and MEGA2 version 4.0 [51, 52]. Bootstrap values were assembled on 500 replicates. Genetic distances and pairwise % identity/similarity matrix (percentage of bases/ residues which are identical) were calculated using Geneious software [51]. Additionally, during the comparative analysis, we also calculated the minimum genetic distance and the maximum (%) identity of the sequences. The Bayesian inference (BI) method was performed using the MrBayes v. 3.2.6 plugin in Geneious 6.1.8 [51], following the procedures outlined by Ren et al. [72].

### 2.4. Sequencing and nucleotide sequence accession numbers

Three genes *wsp*, *coxA* and *ftsZ*) were amplified and sequenced from the populations containing *Wolbachia*. Cytochrome c oxidase subunit I (*COI*) and *12S rRNA* genes were also sequenced for molecular identification and barcoding of *C*. *maculatus*. PCR products were run through an ABI 3730 DNA analyzer in both directions. After manual processing, the original sequences were assembled using the GP 2024 v R11 software. Nine sequences of five genes were deposited to the Genbank database under accession numbers (*12S rRNA;* PP209111, OQ934051, PP212965), (*COI*: PP188367, PP177610, PP188097), (*coxA*: PP331220), (*ftsZ*: PQ375108) and (*wsp*: PQ375109).

### 2.5. Statistical analysis

Statistical significance of the differences in populations between different groups was evaluated using Chi*-*squared tests in R 4.4.1 [53]. The null hypothesis (H_0_) presumed that the variables were independent, and the significance level was accustomed at 0.01. Percentage of the amplifications was calculated by using the formula (Amplified individuals (n)/total tested (N_2_) × 100. Significance of infection frequencies was assessed using univariate analysis of variance. This was followed by a post hoc Tukey’s HSD multiple comparison analysis at a significance level of 0.05 conducted by using the R software [53].

## 3. Results

### 3.1. Molecular identification and phylogenetics of the host *Callosobruchus maculatus*

The PCR with the *12S rRNA* and cytochrome c oxidase subunit I (*COI*) genes using 116 samples from various locations resulted in an overall amplification of 94.85% and 78.48% for *C*. *maculatus* populations, respectively (S1 Table).

The amplification rates varied from 70.83% to 100% for both tested genes. The highest amplification rates for the *12S rRNA* gene were observed in the populations of Faisalabad and Peshawar, followed by Lahore, Multan, and Hyderabad. On the other hand, the highest amplification rates for the cytochrome oxidase subunit I (*COI*) gene were observed in Lahore, followed by Multan, Peshawar, Hyderabad, and Faisalabad (F_1,9_ = 18.59, p < 0.0026). The sequences of *12S rRNA* (279–360 bp) and *COI* (554–570 bp) were obtained. The retrieved sequences from the present study based on both genes were compared with already published sequences of *C*. *maculatus* from GenBank and witin the same gene.

**Fig 1:**
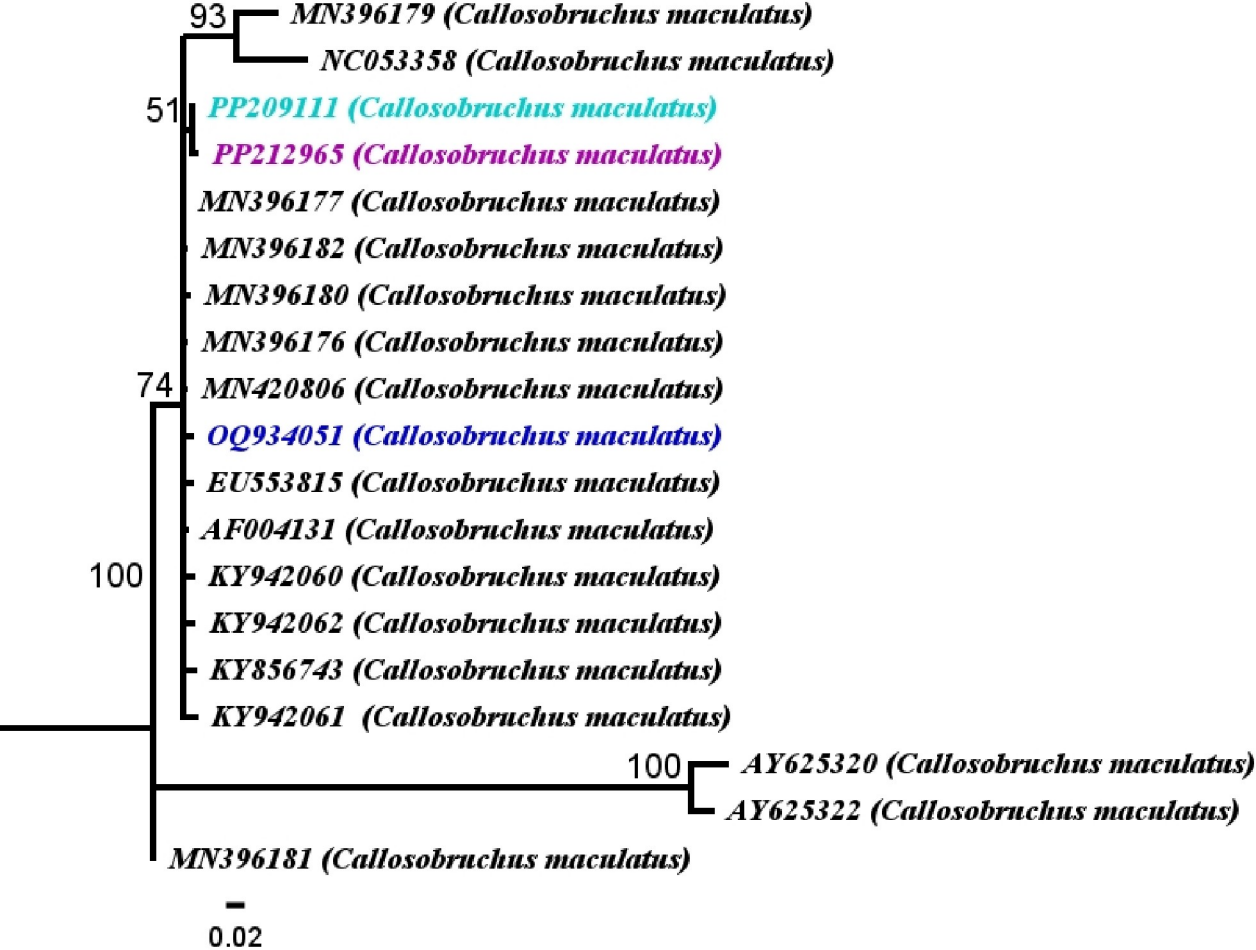
Phylogenetic analysis of *Callosobruchus maculatus* relationships inferred from *12S rRNA* sequences based on Neighbor*-*Joining (NJ) analysis method (Tamura*-*Nei substitution model). Bootstrap digits were constructed on 500 replicates (> 50%). Accession numbers are mentioned with the sequence.

According to the analysis of the *12S rRNA* gene sequences, it was found that the sequences retrieved closely resemble the *12S rRNA* sequences of *C*. *maculatus* from the GenBank data. These sequences were compared to sixteen other *C*. *maculatus* sequences from different origions within the same gene from the GenBank, and neighbor*-*joining (NJ) trees phylogeny were constructed. The genetic distances and % identity matrix showed that the studied populations (PP209111, PP212965, OQ934051) of *C*. *maculatus* were clustered together with the *C*. *maculatus* sequences in the GenBank data. The population (PP209111) is closely related with the populations (India, KY856743, GD 0.02, identity 99.7%). Whereas (PP212965) was closely clustered with (France, AF004131, GD 0.02, identity 99.33%) Further thethe population (OQ934051) was closely clustered with the population ((India, KY856743, GD 0.02, identity 99.64%) presented in (Fig 1; S3 Table).

**Fig 2:**
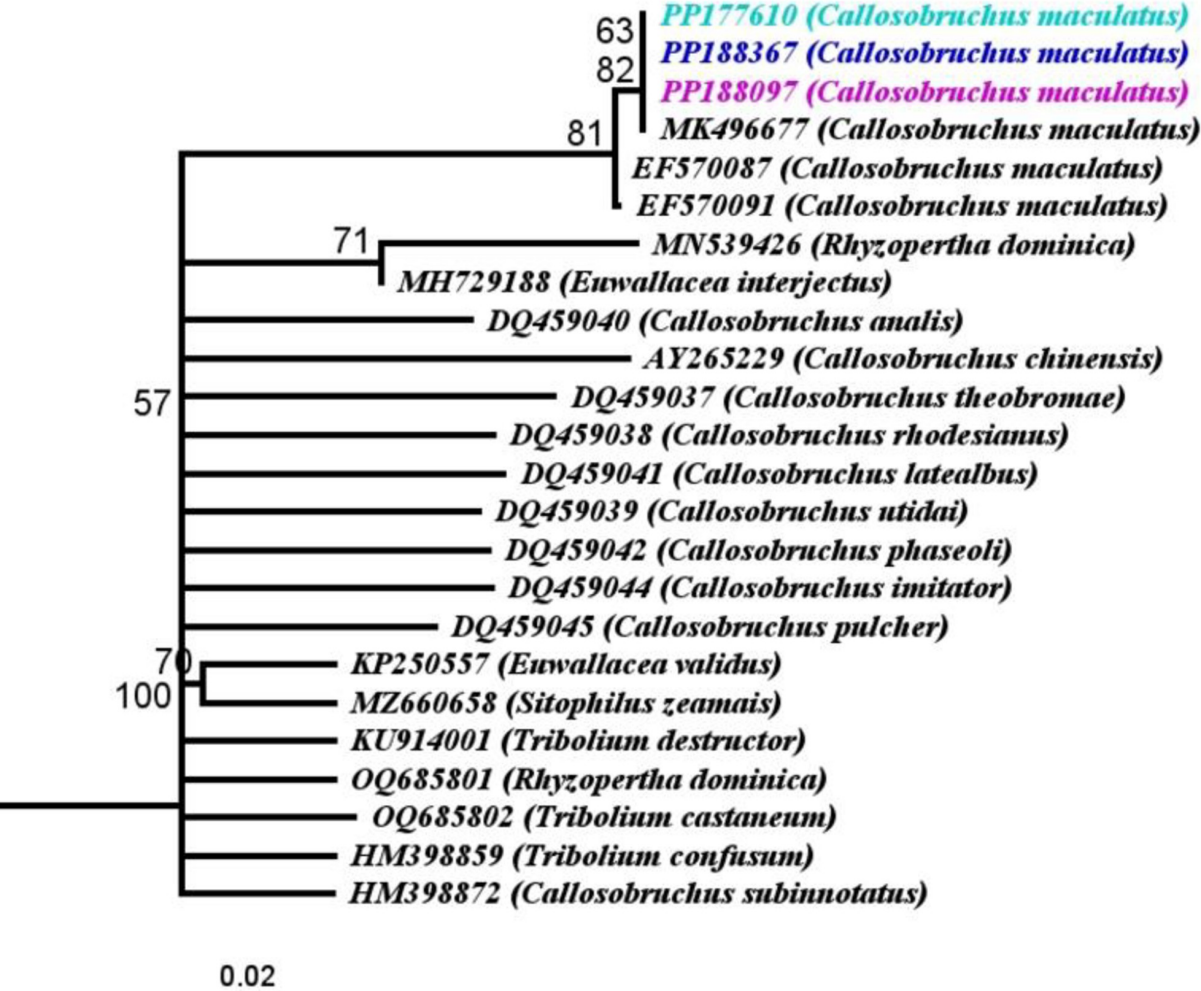
Phylogenetic analysis of *Callosobruchus maculatus* relationships inferred from Cytochrome Oxidase I (*COI*) sequences based on Neighbor*-*Joining (NJ) analysis method (Tamura*-*Nei substitution model). Bootstrap digits were constructed on 500 replicates (> 50%). Accession numbers are mentioned with the sequence.

The sequence analysis based on *COI* gene exhibited that studied sequences were exactly resembled the *COI* sequences of *C*. *maculatus* from the GenBank data. These sequences were compared to the twenty one *C*. *maculatus* and other Coleoptera sequences of different countries within the same gene from the GenBank. Neighbor*-*joining (NJ) trees phylogeny were constructed. The investigated statistics (genetic distances, % identity matrix) exhibited that the studied populations (PP177610, PP188367, PP188097) of *C*. *maculatus* were exactlt clustedred with the *C*. *maculatus* population from other countries from GenBank data. These populations were closely related with the population (India, MK496677, GD 0.01, identity 99.12–100%) presented in (Fig 2; S4 Table).

### 3.2. Screening of *Wolbachia* in *Callosobruchus maculatus*

The screening of 116 samples resulted 38.41% (*wsp*), 33.18% (*coxA*), 31.59% (*ftsZ*) were found positive for *Wolbachia* infection (S1 Table). The infection rates ranged from 18.18% to 54.16% across all three tested *Wolbachia* genes. The highest infection rates were observed in populations of Faisalabad, followed by Lahore, Hyderabad, Multan, and Peshawar (Chi*-*squared test: 3.968, p*-*value < 0.0464).

**Fig 3:**
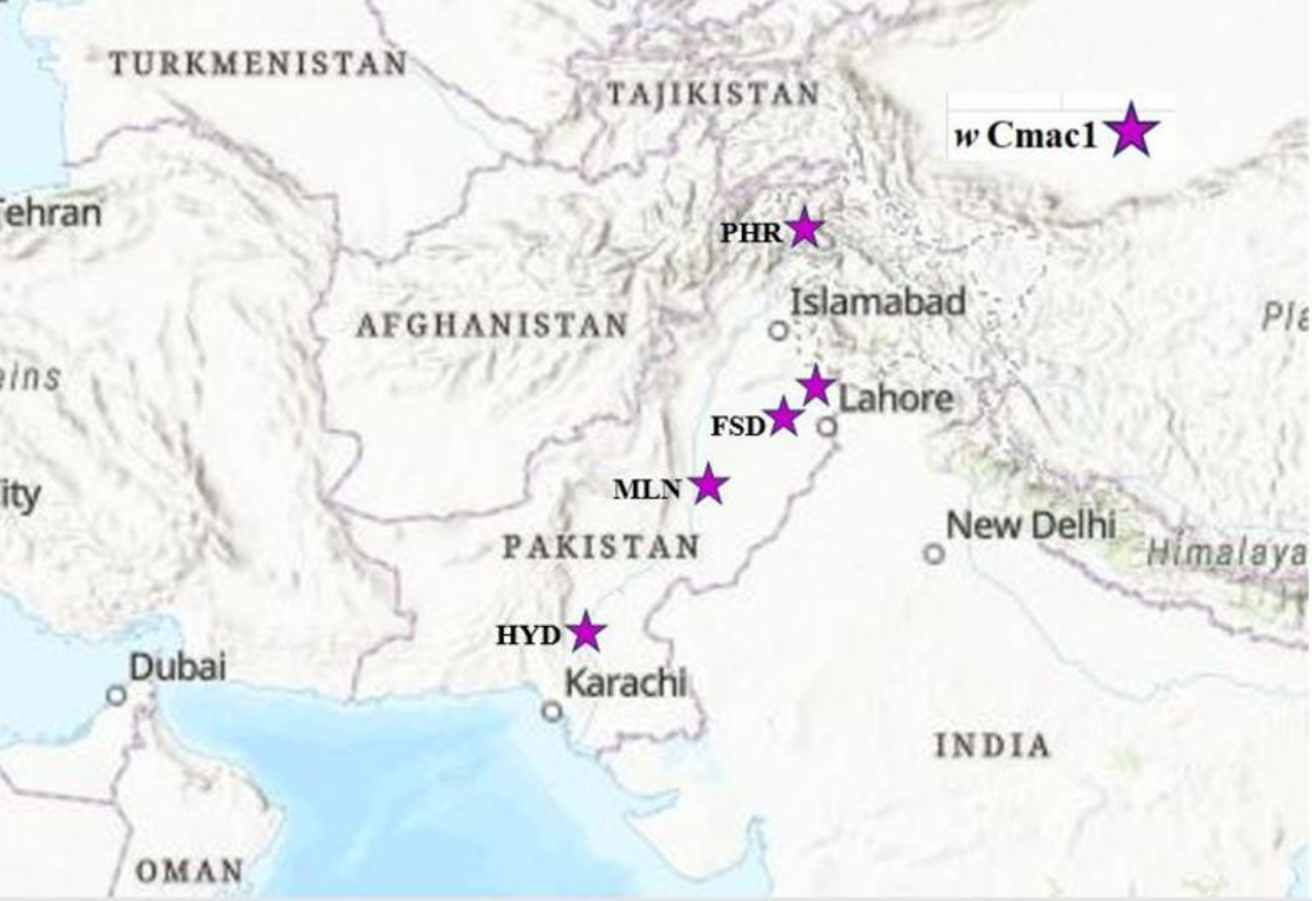
Map showing collection sites and distribution of *Wolbachia* strain in *Callosobruchus maculatus*. Map created with (ArcGIS; Esri, Tom Tom, Garmin, FAO, NOAA, USGS). Locations: Faisalabad (FSD), Multan (MLN), Peshawar (PHR), Hyderabad (HYD) and Lahore.

**Fig 4:**
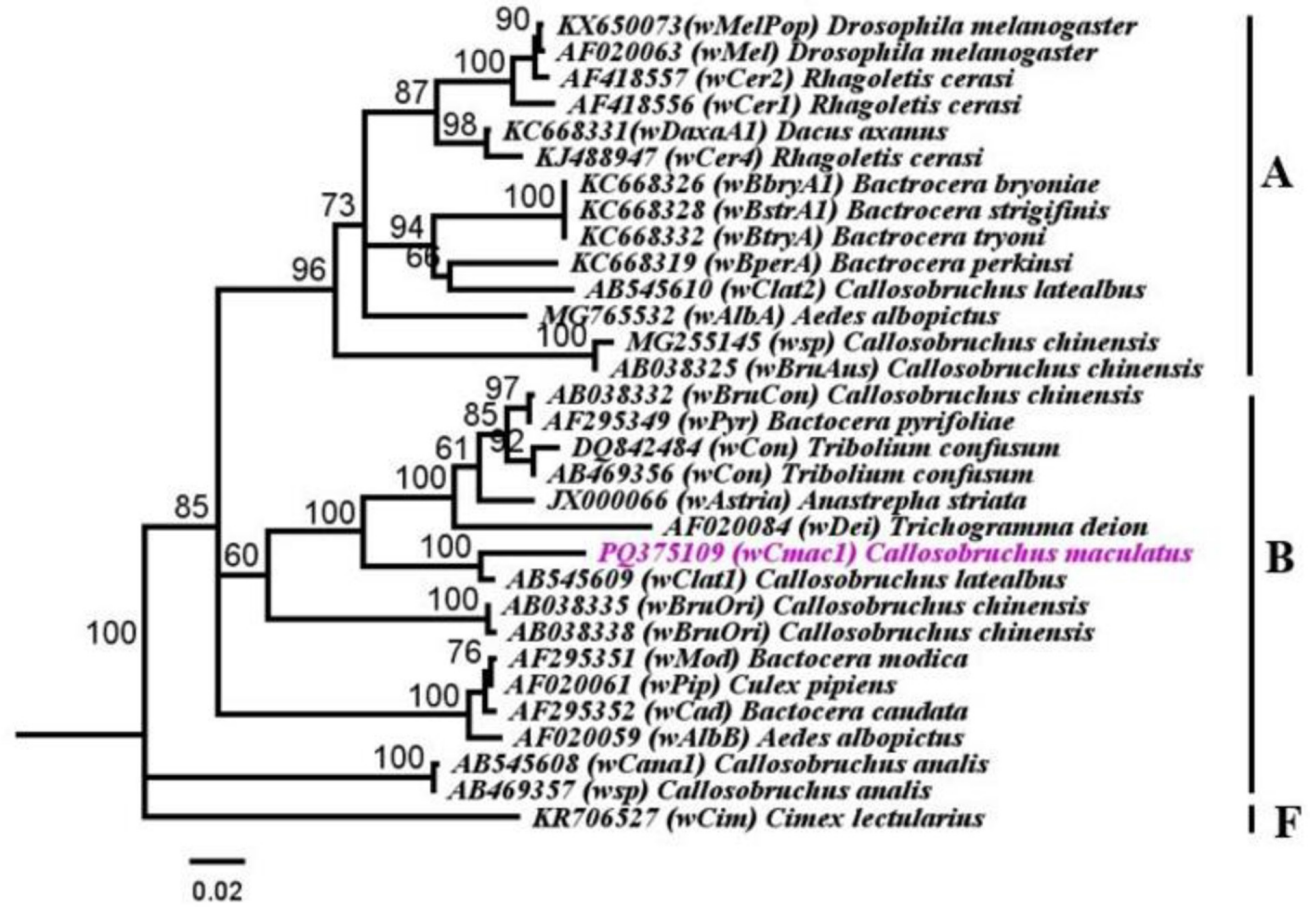
Phylogenetic analysis of *Wolbachia* strain (*w*Cmac1) of *Callosobruchus maculatus* and other *Wolbachia* strains relationships inferred from *wsp* dataset PQ375109 based on Neighbor*-*Joining (NJ) analysis method (Tamura*-*Nei substitution model). Bootstrap digits were constructed based on 500 replicates (> 50%) shown adjacent to the branches of clades. Accession numbers and *Wolbachia* supergroups are mentioned in the sequences. KR706527 *Cimex lectularius* (*w*Cim) used as outgroup.

A phylogenetic analysis was conducted to compare the identified *Wolbachia* strain in *C*. *maculatus* with 30 other strains, including those from different origins documented in GenBank. The genetic identity of the *Wolbachia* in *C*. *maculatus* was determined by aligning and analyzing the *wsp* gene sequences with *Wolbachia* sequences from 14 A, 15 B, and 01 F supergroups from various origins. The analysis of the *wsp* gene sequences revealed the presence of one distinct *Wolbachia* strain clustered in supergroup B (Fig 3).The sequence (PQ375109) revealed the lowest genetic distance within supergroup B with accession numbers AB545609 (GD 0.04; identity 93.63%) originated from Japan. (Figs 3 and 4; S5 Table).

The prevalence of *Wolbachia* in *C*. *maculatus* populations and comparison with other *Wolbachia* strains from different *Wolbachia* harboring populations of various origins were accomplished based on two MLST gene (*coxA* and *ftsZ*) data.

**Fig 5:**
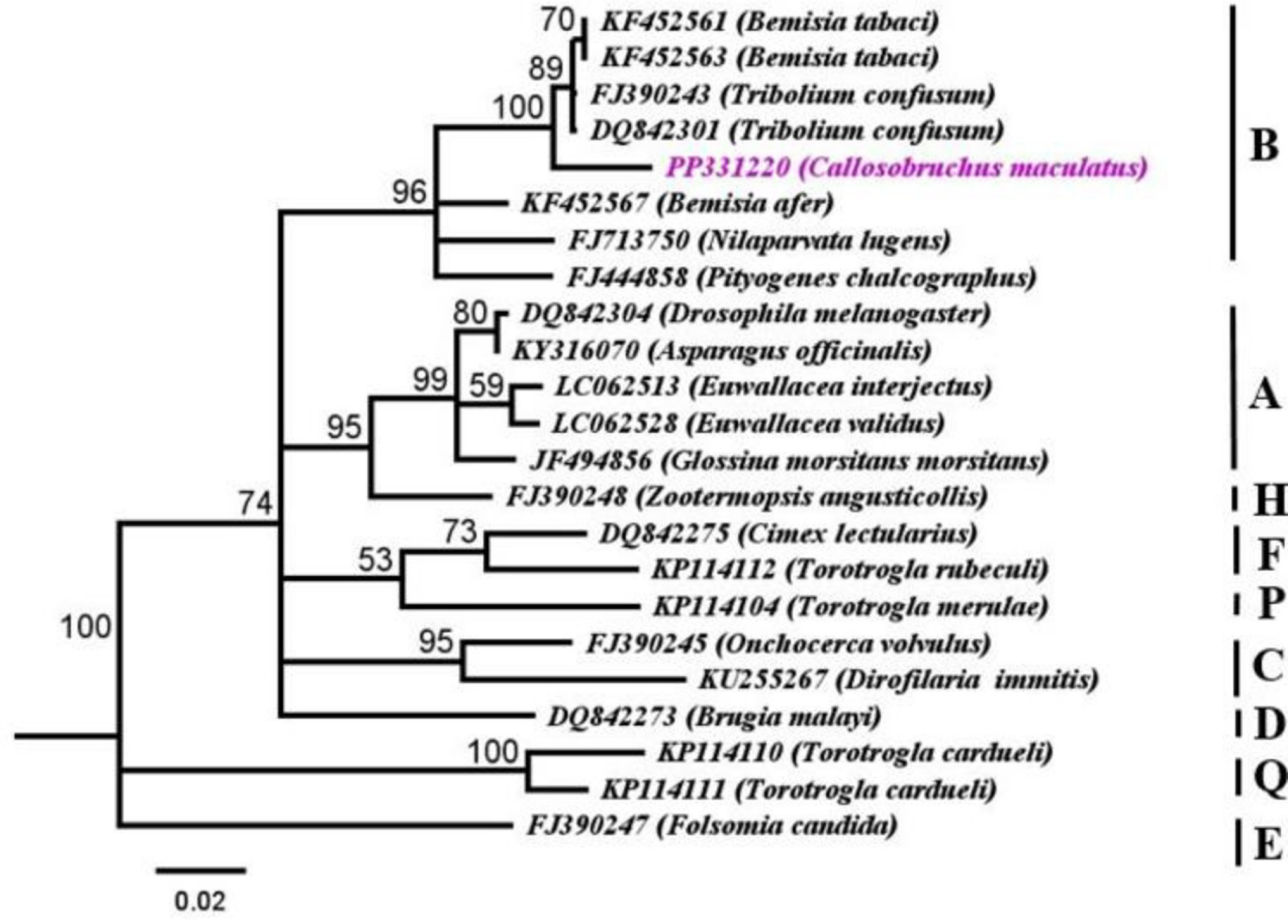
Phylogenetic analysis of *Wolbachia* strain of *Callosobruchus maculatus* relationships inferred from *coxA* (MLST) PP331220 dataset based on Neighbor*-*Joining (NJ) analysis method (Tamura*-*Nei substitution model). Bootstrap digits were constructed based on 500 replicates (> 50%) shown adjacent to the branches of clades. Accession numbers and *Wolbachia* supergroups are mentioned in the sequences. FJ390247 *Folsomia candida* used as outgroup.

Samples of *C*. *maculatus* from various populations were examined for *Wolbachia* infection by analyzing the *coxA* gene. A phylogenetic analysis was conducted to compare the identified *Wolbachia* strains with 22 other strains from different origins documented in GenBank. The analysis showed that the strain identified as PP331220 belongs to supergroup B with moderate to high bootstrap values (53–100%). Comparison of the *Wolbachia* strains exhibited that studied strain named (*w*Cmac1) from *C*. *maculatus* was closely related with (DQ832301, GD 0.03, identity 97.51%, *Tribolium confusum*) and (FJ390243, GD 0.03, similarity 97.51%, *T*. *confusum*) presented in (Fig 5; S6 Table).

**Fig 6:**
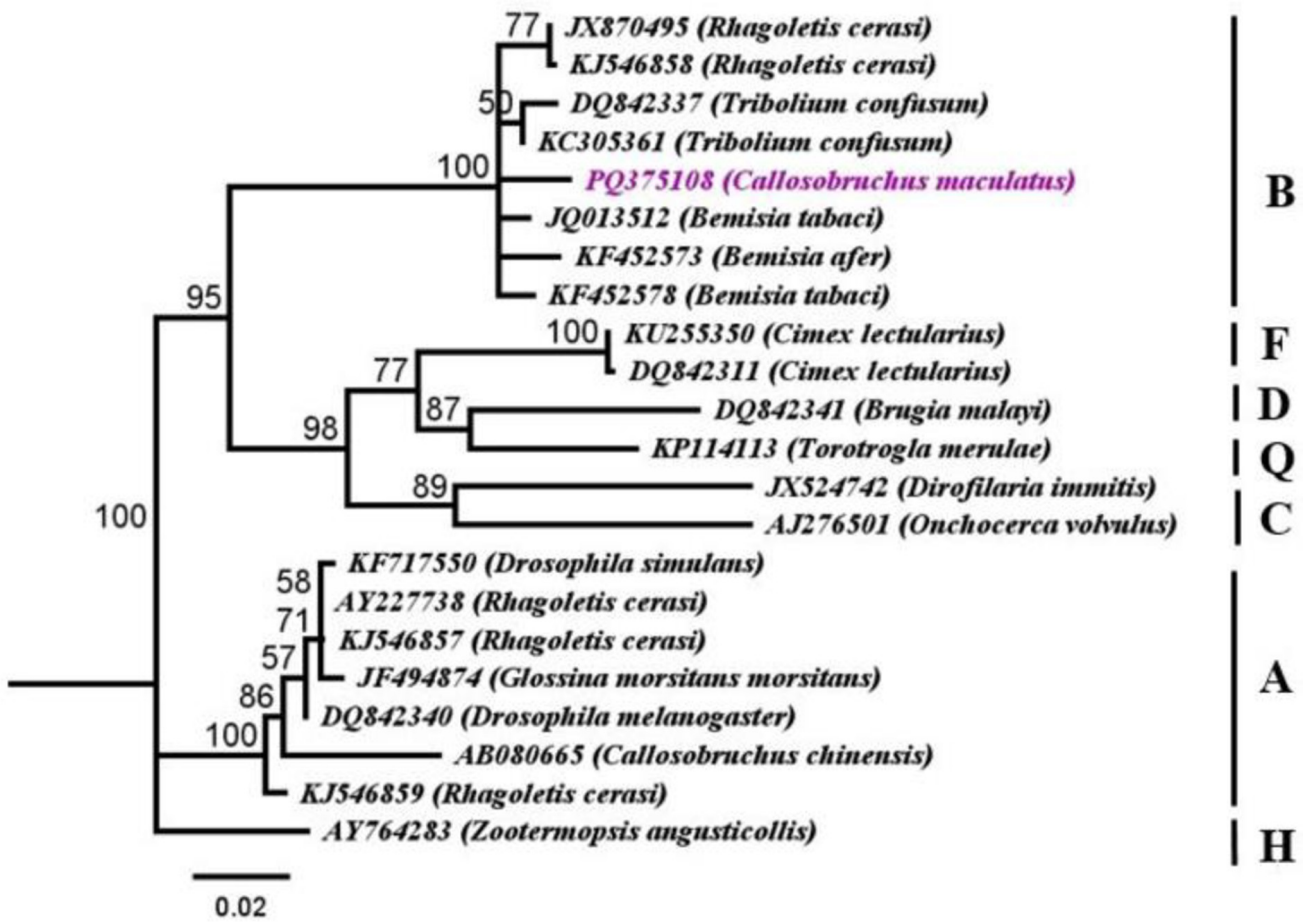
Phylogenetic analysis of *Wolbachia* strain of *Callosobruchus maculatus* relationships inferred from *ftsZ* (MLST) PQ375108 dataset based on Neighbor*-*Joining (NJ) analysis method (Tamura*-*Nei substitution model). Bootstrap digits were constructed based on 500 replicates (> 50%) shown adjacent to the branches of clades. Accession numbers and *Wolbachia* supergroups are mentioned in the sequences. AY764283 *Zootermopsis angusticollis* used as outgroup.

The samples of *C*. *maculatus* were tested for *Wolbachia* infection using the *ftsZ* one of the five houskeeping (MLST) genes. The comparision analysis with 21 other *Wolbachia* sequences within the same gene from different origins documented in GenBank revealed that the strain (PQ375108) belongs to supergroup B (Fig 6) with moderate to high bootstrap values (52–100%). Furthermore, analysis exhibited that studied strain from *C*. *maculatus* was closely related with (KC305361, GD 0.02, identity 99.39%, *Tribolium confusum*) presented in (Fig 6; S7 Table). Based on the analysis of three *Wolbachia* genes the identified strain named *w*Cmac1 according to *Wolbachia* nomenclature as it was found to be distinct from other *C*. *maculatus* strains.

## 4. Discussion

Molecular DNA based identification using mitochondrial genes is an effective technique for identifying insect species and distinguishing between closely related species [32, 54, 55]. It is more reliable than conventional morphological identification, which can be time consuming and technically challenging. Cytochrome oxidase subunit I (*COI*) based DNA barcoding has been useful in identifying many cryptic and sibling species [56, 57]. It is therefore considered that molecular identification techniques are more reliable than conventional methods of morphological identification [58].

Research has shown that DNA*-*based barcoding using mitochondrial genes is a quick and accurate method for identifying arthropod specimens [32, 34, 58]. Both approaches can be utilized depending on the available resources and the environment. In the present study molecular identification of *C*. *maculatus* was successfully demonstrated using both *COI* and *12S rRNA* genes.

*Wolbachia* is endosymbiotic bacteria that is widely present in various insects, nematodes, mites, springtails, spiders, crustaceans, bivalves, and tardigrades [24, 59–65]. It has a close relationship with mitochondrial genotypes within species and is typically transmitted vertically from one generation to the next. However, horizontal transfer may also play a role in its spread within a species. For example, *Wolbachia* was found to spread horizontally when infected and uninfected *Trichogramma* wasp larvae shared the same host [66]. Additionally, hymenopteran parasitoids of *Drosophila* species acquired *Wolbachia* through frequent horizontal transmission [67].

*Wolbachia*, a bacterium primarily transmitted maternally in arthropods [29], can manipulate the host’s reproductive system through cytoplasmic incompatibility (CI) and colonize new mitochondrial lineages [24, 60, 68]. Maternal transmission occurs primarily through the cytoplasm of eggs [29]. There are recognized monophyletic lineage groups labeled A to R [43, 69] with supergroup G present in Australian spiders [70].

The presence of *Wolbachia* in insects has been identified using different marker genes, but the reliability of these markers varies depending on the insect species [60, 71]. The *wsp* gene evolves more quickly among various *Wolbachia* strains, making PCR amplification less reliable when using current primer pairs [39, 72]. Although the *wsp* method is commonly used for diagnosing A and B group *Wolbachia* [49, 73] its potential for strain characterization is limited due to frequent recombination [74]. Nevertheless, *wsp* remains one of the most polymorphic markers available for detecting *Wolbachia* diversity and is widely used as the starting point for large screening experiments. To address the frequent recombination issue of *wsp* gene, two genes of multilocus sequence typing (MLST) based on five housekeeping genes has applied to explore *Wolbachia* diversity. MLST is a reliable tool for studying population genetics and molecular evolution, helping to minimize errors caused by frequent recombination observed in *wsp* [39]. Positive amplification of *Wolbachia* in *C*. *maculatus* populations was found based on *wsp*, along with two genes (*coxA* and *ftsZ*) of MLST, providing preliminary evidence of *Wolbachia* infection in *C*. *maculatus*. The present identified strain belonging to supergroup B, and this strain is also distinct from the already published *Wolbachia* strains in *Callosobruchus* species.

Kajtoch and Kotásková [23] thoroughly reviewed *Wolbachia* in Coleopteran hosts, briefing on a single *Wolbachia* strain in 43 species, two strains in 10 species, and multiple infections in 9 species. Previous research has also looked into the *Wolbachia* strains in *Callosobruchus* species. For example, Kondo et al. [75] examined the *wsp* and *ftsZ* genes to investigate *Wolbachia* in *C*. *chinensis*, *C*. *analis*, and *C*. *latealbus*. In another study, Kageyama et al. [76] used *wsp* genes to analyze the *Wolbachia* strains in *C*. *analis* and *C*. *chinensis*. In Japanese populations of *C*. *chinensis*, the *Wolbachia* strains *w*BruCon and *w*BruOri were found to have almost 100% infection frequencies and to cause cytoplasmic incompatibility [77]. Furthermore, Kondo et al. 76] also reported the 100% infection frequencies of *w*Cana1 in *C*. *analis* and *w*Clat2 in *C*. *latealbus*. Two distinct *Wolbachia* strains have been identified in *C*. *analis*, (non-CI-inducing *w*Cana1 and CI-inducing *w*Cana2. Field-collected *C*. *analis* individuals were either singly infected with *w*Cana1 or double infected with *w*Cana1 and *w*Cana2. [75, 78, 79].

No previous literature was found on *Wolbachia* infection in *C*. *maculatus*. Therefore, by comparing the available data of *Callosobruchus* species it is stated that current findings on *Wolbachia* strains in *C*. *maculatus* populations are new and have not been characterized before using a multiple gene amplification approach.

Mitochondrial DNA and *Wolbachia* have the same cytoplasmic pathway of transmission. The present research revealed the phylogenetic interpretation of cowpea weevil with *Wolbachia* amplification and the effects of the host mitochondrial DNA evolution and diversification [80]. In the current investigation, low titer *Wolbachia* density were found with the all three *Wolbachia* genes. Previous studies have found that *Wolbachia* can be hard to detect due to low bacterial densities [71]. It seems that all species in a diverse group of insects are infected with *Wolbachia*, but the infections are sometimes at low density or only affect some individuals within a population. Bordenstein et al. [81] suggested that low *Wolbachia* densities might be caused by high densities of its associated bacteriophage.

Molecular phylogenetic analysis revealed that *C*. *maculatus* harbors a *Wolbachia* bacterium from supergroup B, which is one of the identified supergroups of arthropods [24]. This distinct *Wolbachia* lineage in *C*. *maculatus* populations was verified by analyzing three *Wolbachia* gene sequences with strong bootstrap values. Additionally, various types of endosymbiotic microorganisms have also been found in related Coleopteran families, such as Chrysomelidae, Curculionidae, and Rhynchophoridae [23].

This is the first report about the presence of *Wolbachia* in *C*. *maculatus* populations. Previous research suggested that *Wolbachia* was also identified in three species of the genus *Callosobruchus* [75, 76]. These findings suggest that it is likely that *Wolbachia* was developed by a common ancestor of *C*. *maculatus*, probably from either a phylogenetically distinct arthropod or an unevaluated bruchid carrier. The evolutionary history of *Wolbachia* may have occurred through interspecific transmissions [82].

It is widely believed that *Wolbachia* is transmitted vertically to the next generation of hosts. This is because it is commonly found in reproductive tissues. However, recent research has shown that *Wolbachia* can be present in various tissues throughout the host [83], which is consistent with our findings that we were able to detect *Wolbachia* in the genomic DNA of the entire insect, not just the reproductive tissues. Previous studies have reported the rate of *Wolbachia* infection in different insect populations [84, 85], which is influenced by factors such as the cost of infection to the host, the effectiveness of vertical transmission, the initial frequency of infection, and the degree of cytoplasmic incompatibility [84, 86].

To better understand the factors responsible for complete septicity in *C*. *maculatus*, it is important to explore certain elements in depth. In order to achieve this, we amplified two mitochondrial genes for host *C*. *maculatus* and three *Wolbachia* genes for the endosymbiotic bacteria. The study of *C*. *maculatus* provides efficient approaches to comprehend the interface and dynamics between *Wolbachia* and its host insect. However, in the future, we will analyze the other *Wolbachia* genes to study the phylogenetic alterations of *Wolbachia* in *C*. *maculatus* from different geographical populations.

*Wolbachia* manipulates its host’s biology through various means, including (CI) cytoplasmic incompatibility [24]. Melanic mutations cause a decline in host fitness [78], and intraspecies variation in cytoplasmic incompatibility intensity also plays a role in host manipulation [79]. Neverthless, the incompatible insect technique (IIT) uses *Wolbachia* to induce conditional sterility in mass-reared infected males crossed with wild females, reducing the target population over time. Studies have shown that IIT effectively controls insect disease vectors and agricultural pests [25, 26, 28].

This is the preliminary study which provides new insights into *Wolbachia* infection in *C*. *maculatus* populations, which has been rarely studied. Factors affecting the symbiotic relationship between arthropods and their symbionts include the cost of infection for hosts, spread of symbionts between insects, and environmental factors like temperature and natural enemies [87, 88]. Excessive use of pesticides, pollution, and changes in environmental factors significantly impact the decrease in mitochondrial DNA [89–91]. The low prevalence of *Wolbachia* in *Callosobruchus* species may be due to high temperatures in tropical and temperate regions [92]. Future studies should consider these factors as they may significantly affect *Wolbachia* diversity and focus.

## 5. Conclusions

The results of the present study suggest that *Wolbachia* infection has been found in the populations of *C*. *maculatus*. The mitochondrial phylogenetic analysis of the host species *C*. *maculatus* populations correctly revealed the molecular identification and clustered with the GenBank data from various origins of the same host and the same gene. The screening of *Wolbachia* in *C*. *maculatus* was accomplished through multiple gene analysis, resulting in the identification of one new strain named *w*Cmac1, belonging to supergroup B. This study will help us understand the dynamics of *Wolbachia* in *C*. *maculatus* and develop effective approaches for pest management.

## Supporting information

S1 Table Samples collections, locations and screening of the *Callosobruchus maculatus*. (XLSX)

S2 Table Primers used, PCR details and obtained products of *12S rRNA*, mitochondrial (*CO1*), *wsp* and (MLST) genes *coxA* and *ftsZ*. (XLSX)

S3 Table Pairwise genetic distance and percent identity values based on the *12S rRNA* gene sequences with GenBank data. (XLSX)

S4 Table Intra and inter specific analysis of pairwise genetic distance and percent identity values based on *COI* gene sequences with GenBank data. (XLSX)

S5 Table Intra and inter specific analysis of pairwise genetic distance and percent identity values based on *wsp* gene sequences with GenBank data. (XLSX)

S6 Table Intra and inter specific analysis of pairwise genetic distance and percent identity values based on *coxA* gene (MLST) and comparison with GenBank data. (XLSX)

S7 Table Intra and inter specific analysis of pairwise genetic distance and percent identity values based on *ftsZ* gene (MLST) and comparison with GenBank entries. (XLSX)

## References

[1] Zhao N, Jiao K, Chiu YH, Wallace TC. Pulse Consumption and Health Outcomes: A Scoping Review. Nutrients. 2024; 16(10):1435. doi: 10.3390/nu16101435 38794673 PMC11124391

[2] Tenorio TA, Kyriakopoulou KE, Suarez-Garcia E, van den Berg C, van der-Goot AJ. Understanding differences in protein fractionation from conventional crops, and herbaceous and aquatic biomass—consequences for industrial use. Trends in Food Science & Technology. 2018; 71: 235–245.

[3] Stagnari F, Maggio AA, Galieni MP. Multiple benefits of legumes for agriculture sustainability: an overview. Chemical and Biological Technologies in Agriculture. 2017; 4: 1–13.

[4] Kalpna Hajam YA, Kumar R. Management of stored grain pest with special reference to *Callosobruchus maculatus*, a major pest of cowpea: A review. Heliyon. 2022; 8 (1): e08703, 10.1016/j.heliyon.2021.e08703. 35036600 PMC8749198

[5] Bouchard P, Andrew B, Smith T, Douglas H, Gimmel ML, Brunke AJ, et al. Biodiversity of Coleoptera. Insect Biodiversity: Science and Society. Vol I: Second Edition. Edit. Robert GF, Peter HA. 2017.

[6] Grove SJ, Stork NE. An inordinate fondness for beetles. Invertebrate Systematics. 2000; 14: 733–739.

[7] Adamski Z, Bufo SA, Chowanski S, Falabella P, Lubawy J, Marciniak P, et al. Beetles as Model Organisms in Physiological, Biomedical and Environmental Studies—A Review. Frontiers in Physiology. 2019; 10: 319. doi: 10.3389/fphys.2019.00319 30984018 PMC6447812

[8] Sarwar M. Assessment of resistance to the attack of cowpea weevil *Callosobruchus maculatus* (Fabricius) in chickpea genotypes on the basis of various parameters during storage. Songklanakarin Journal of Science and Technology. 2012; 34 (3), 287–291.

[9] Tuda M, Kagoshima K, Toquenaga Y, Arnqvist G. Global genetic differentiation in a cosmopolitan pest of stored beans: effects of geography, host plant usage and anthropogenic factors. PLoS One. 2014; 9(9):e106268. doi: 10.1371/journal.pone.0106268 25180499 PMC4152179

[10] Tuda M. Applied evolutionary ecology of insects of the subfamily Bruchinae (Coleoptera: Chrysomelidae). Applied Entomology and Zoology. 2007; 42: 337–346. doi: 10.1303/aez.2007.337

[11] Tuda M, Rönn J, Buranapanichpan S, Wasano N, Arnqvist G. Evolutionary diversification of the cowpea weevil genus *Callosobruchus* (Coleoptera: Bruchidae): Traits associated with stored*-*product pest status. Molecular Ecology. 2006; 15: 3541–3555. doi: 10.1111/j.1365<italic>-</italic>294X.2006.03030.x 17032256

[12] Nisar MS, Haq IU, Ramzan H, Aljedani DM, Qasim M, Islam W, et al. Screening of different legumes for the developmental preference of *Callosobruchus maculatus* (Bruchidae: Coleoptera). International Journal of Tropical Insect Science. 2021; 41: 3129–3136. 10.1007/s42690-021-00507-6.

[13] Hagstrum DW, Phillips TW. Evolution of stored*-*product entomology: protecting the world food supply. Annual Review of Entomology. 2017; 62:379–397. 10.1146/annurev-ento-031616-035146 28141965

[14] Hamzavi F, Naseri B, Hassanpour M, Razmjou J, Golizadeh A. Biology and life table parameters of *Callosobruchus maculatus* (F.) on *Vigna unguiculata* (L.) Walp. fertilized with some mineral- and bio-fertilizers. Journal of Stored Products Research. 2022; 97: 101978, 10.1016/j.jspr.2022.101978.

[15] Haddi K, Jumbo LV, Costa M, Santos M, Faroni L, Serrão J, et al. Changes in the insecticide susceptibility and physiological trade offs associated with a host change in the bean weevil *Acanthoscelides obtectus*. Journal of Pest Science. 2018; 91(1): 459–468. doi: 10.1007/s10340-017-0860-1

[16] Lopes LM, Sousa AH, Santos VB, Silva GN, Abreu AO. Development rates of *Callosobruchus maculatus* (Coleoptera: Chrysomelidae) in landrace cowpea varieties occurring in southwestern Amazonia. Journal of Stored Products Research. 2018; 76: 111–115. doi: 10.1016/j.jspr.2018.01.008

[17] Massango H, Faroni L, Haddi K, Heleno F, LO VJ, Oliveira E. Toxicity and metabolic mechanisms underlying the insecticidal activity of parsley essential oil on bean weevil, *Callosobruchus maculatus*. Journal of Pest Science. 2017; 90(2): 723–733. doi: 10.1007/s10340-016-0826-8

[18] Kang JK, Pittendrigh BR, Onstad DW. Insect resistance management for stored product pests: a case study of cowpea weevil (Coleoptera: Bruchidae). Journal of Economic Entomology. 2013; 106(6): 2473–2490. doi: 10.1603/ec13340 24498750

[19] Iturralde-García RD, Borboa-Flores J, CincoMoroyoqui FJ, Riudavets J, Del Toro-Sánchez CL, Rueda-Puente EO, et al. Effect of controlled atmospheres on the insect *Callosobruchus maculatus* (Fab) in stored chickpea. Journal of Stored Products Research. 2016; 69: 78–85. doi: 10.1016/j.jspr.2016.06.004

[20] Baumann P, Moran NA. Non*-*cultivable microorganisms from symbiotic associations of insects and other hosts. Antonie van Leeuwenhoek.1997; 72: 38–48. 10.1023/a:1000239108771 9296262

[21] Inaki IO, Woolfit M, Rances E, Duplouy A, O’Neill SL. A simple protocol to obtain highly pure *Wolbachia* endosymbiont DNA for genome sequencing. Journal of Microbiological Methods. 2011; 84(1): 134–136. 21047535 10.1016/j.mimet.2010.10.019

[22] Hilgenboecker K, Hammerstein P, Schlattmann P, Telschow A, Werren JH. How many species are infected with *Wolbachia*? A statistical analysis of current data. *FEMS* Microbiology Letters. 2008; 281: 215–220 doi: 10.1111/j.1574<italic>-</italic>6968.2008.01110.x 18312577 PMC2327208

[23] Kajtoch L, Kotásková N. Current state of knowledge on *Wolbachia* infection among Coleoptera: a systematic review. PeerJ. 2018; 6: e4471. doi: 10.7717/peerj.4471 29568706 PMC5846457

[24] Werren JH, Baldo L, Clark ME. *Wolbachia*: master manipulators of invertebrate biology. Nature Reviews Microbiology. 2008; 6: 741–751. 18794912 10.1038/nrmicro1969

[25] Zabalou S, Riegler M, Theodorakopoulou M, Stauffer C, Savakis C, Bourtzis K. *Wolbachia-*induced cytoplasmic incompatibility as a means for insect pest population control. Proceedings of the National Academy of Sciences, USA. 2004b; 101: 15042–15045. doi: 10.1073/pnas.0403853101 15469918 PMC524042

[26] Zabalou S, Apostolaki A, Livadaras I, Franz G, Robinson A.S., Savakis C., et al. Incompatible insect technique: incompatible males from a *Ceratitis capitata* genetic sexing strain. Entomologia Experimentalis et Applicata. 2009; 132: 232–240.

[27] Mateos M, Montoya MH, Lanzavecchia SB, Conte C, Guillén K, Morán-Aceves BM, et al. *Wolbachia* pipientis Associated With Tephritid Fruit Fly Pests: From Basic Research to Applications. Frontiers in Microbiology. 2020;11: 1080. doi: 10.3389/fmicb.2020.0108 32582067 PMC7283806

[28] Xu X, Ridland PM, Umina PA, Gill A, Ross PA, Pirtle E, et al. High Incidence of Related *Wolbachia* across Unrelated Leaf-Mining Diptera. Insects. 2021; 12(9), 788. 10.3390/insects12090788. 34564228 PMC8465256

[29] Schuler H, Köppler K, Daxböck-Horvath S, Rasool B, Krumböck S, Schwarz D, et al. The hitchhiker’s guide to Europe: the infection dynamics of an ongoing *Wolbachia* invasion and mitochondrial selective sweep in *Rhagoletis cerasi*. Molecular Ecology. 2016; 25(7):1595–609. doi: 10.1111/mec.13571 26846713 PMC4950298

[30] McMeniman CJ, Lane RV, Cass BN, Fong AWC, Sidhu M, Wang YF, et al. Stable introduction of a life*-*shortening *Wolbachia* infection into the mosquito *Aedes aegypti*. Science. 2009; 323: 141–144. 19119237 10.1126/science.1165326

[31] Qasim M, Baohua W, Zou H, Lin Y, Dash CK, Bamisile BS, et al. Phylogenetic relationship and genetic diversity of citrus psyllid populations from China and Pakistan and their associated Candidatus bacterium. Molecular Phylogenetics and Evolution, 2018; 126: 173–180, doi: 10.1016/j.ympev.2018.04.028 29684596

[32] Simon C, Frati F, Beckenbach A, Crespi B, Liu H, Flook P. Evolution, weighting, and phylogenetic utility of mitochondrial gene sequences and a compilation of conserved polymerase chain reaction primers. Annals of the Entomological Society of America. 1994; 87(6): 651–701.

[33] Cariou M, Duret L, Charlat S. The global impact of *Wolbachia* on mitochondrial diversity and evolution. Journal of Evolutionary Biology. 2017; 30: 2204–2210. 28977708 10.1111/jeb.13186

[34] Kambhampati S, Smith PT. PCR primers for the amplification of four insect mitochondrial gene fragments. Insect Molecular Biology.1995; 4(4): 233–236. doi: 10.1111/j.1365-2583.1995.tb00028.x 8825760

[35] Kosakyan A, Heger TJ, Leander BS, Todorov M, Mitchell EAD, Lara E. *COI* Barcoding of Nebelid Testate Amoebae (Amoebozoa: Arcellinida): Extensive Cryptic Diversity and Redefinition of the Hyalospheniidae Schultze. Protist. 2012; 163 (3): 415–434. doi: 10.1016/j.protis.2011.10.003 22130576

[36] Braig HR, Zhou WG, Dobson SL, O’Neill SL. 1998. Cloning and characterization of a gene encoding the major surface protein of the bacterial endosymbiont *Wolbachia pipientis*. Journal of Bacteriology.1998; 180(1): 2373–2378. 9573188 10.1128/jb.180.9.2373-2378.1998 PMC107178

[37] Urwin R, Maiden MC. Multilocus sequence typing: a tool for global epidemiology. Trends in Microbiology. 2003; 11(10): 479–487. doi: 10.1016/j.tim.2003.08.006 14557031

[38] Zhu Y, Fournier PE, Eremeeva M, Raoult D. Proposal to create subspecies of *Rickettsia conorii* based on multilocus sequence typing and an emended description of *Rickettsia conorii*. BMC Microbiology. 2005; 5(1): 11. 15766388 10.1186/1471-2180-5-11 PMC1079849

[39] Baldo L, Dunning Hotopp JC, Jolley KA, Bordenstein SR, Biber SA, Choudhury RR, et al. Multilocus sequence typing system for the endosymbiont *Wolbachia pipientis*. Applied and Environmental Microbiology. 2006; 72(11): 7098–7110. 16936055 10.1128/AEM.00731-06 PMC1636189

[40] Paraskevopoulos C, Bordenstein SR, Wernegreen JJ, Werren JH, Bourtzis K. Toward a *Wolbachia* multilocus sequence typing system: discrimination of *Wolbachia* strains present in *Drosophila* species. Current Microbiology. 2006; 53(5): 388–95. doi: 10.1007/s00284-006-0054-1 17036209

[41] Stahlhut JK, Desjardins CA, Clark ME, Baldo L, Russell JA, Werren JH, et al. The mushroom habitat as an ecological arena for global exchange of *Wolbachia*. Molecular Ecology. 2010; 19(9): 1940–1952. 20529071 10.1111/j.1365-294X.2010.04572.x

[42] Bing XL, et al. Diversity and evolution of the *Wolbachia* endosymbionts of *Bemisia* (Hemiptera: Aleyrodidae) whiteflies. Ecology and Evolution. 2014; 13: 2714–2737. 10.1002/ece3.1126 PMC4113295 25077022

[43] Inácio da Silva LM, Dezordi FZ, Paiva MHS, Wallau GL. Systematic Review of *Wolbachia* Symbiont Detection in Mosquitoes: An Entangled Topic about Methodological Power and True Symbiosis. Pathogens. 2021;10: 39. 33419044 10.3390/pathogens10010039 PMC7825316

[44] Babu SR, Raju SVS, Singh PS, Sharma KR. Determination of toxicity of newer insecticide molecules against pulse beetle, *Callosobruchus maculatus* (Fabricius) (Chrysomelidae: Coleoptera) under laboratory conditions. Journal of Experimental Biology and Agricultural Sciences. 2020; 8 (1): 35−40.

[45] Vilela ADO, D’Antonino-Faroni LR, Gomes JL, de Sousa AH, Cecon PR. Allyl isothiocyanate as a fumigant in the cowpea and its effect on the physical properties of the grain. Revista Ciencia Agronomica. 2021; 52 (3): e20207287.

[46] Armijos MJG, Jumbo LOV, D’Antonino-Faroni LR, Oliveira EE, Flores-Fernanda AF, Heleno F, et al. Fumigant toxicity of eugenol and its negative effects on biological development of *Callosobruchus maculatus* L. Ciencia y Agricultura. 2019; 36(1): 5–15.

[47] Arthofer W, Riegler M, Schneider D, Krammer M, Miller WJ, Stauffer C. Hidden *Wolbachia* diversity in field populations of the European cherry fruit fly, *Rhagoletis cerasi* (Diptera, Tephritidae). Molecular Ecology. 2009; 18: 3816–3830. doi: 10.1111/j.1365<italic>-</italic>294X.2009.04321.x 19732336

[48] Thompson JD, Higgins DG, Gibbson TJ. Clustal W: improving the sensitivity of progressive multiple sequence alignment through sequence weighting, position specific gap penalties and weight matrix choice. Nucleic Acids Research.1994; 22(1): 4673. doi: 10.1093/nar/22.22.4673 7984417 PMC308517

[49] Jeyaprakash A, Hoy MA. Long PCR improves *Wolbachia* DNA amplification: *wsp* sequences found in 76% of sixty*-*three arthropod species. Insect Molecular Biology. 2000; 9(4): 393–405. 10971717 10.1046/j.1365-2583.2000.00203.x

[50] Altschul SF, Madden TL, Schaffer AA, Zhang J, Zhang Z, Miller W, et al. Gapped BLAST and PSI*-*BLAST: a new generation of protein database search programs. Nucleic Acids Research.1997; 25(17): 3389–3402. 9254694 10.1093/nar/25.17.3389 PMC146917

[51] Kearse M, Moir R, Wilson A, Stones-Havas S, Cheung M, Sturrock S, et al. Geneious basic: an integrated and extendable desktop software platform for the organization and analysis of sequence data. Bioinformatics. 2012; 28: 1647–9. doi: 10.1093/bioinformatics/bts199 22543367 PMC3371832

[52] Kumar S, Tamura K, Jakobsen IB, Nei M. MEGA2: molecular evolutionary genetics analysis software. Bioinformatics. 2001; 17(12): 1244–1245. doi: 10.1093/bioinformatics/17.12.1244 11751241

[53] Team RC. R: A language and environment for statistical computing. R Foundation for Statistical Computing, Vienna, Austria. 2021; https://www.R-project.org/.

[54] Alajmi R, Abdel-Gaber R, AlOtaibi N. Characterization of the *12S rRNA* Gene Sequences of the Harvester Termite *Anacanthotermes ochraceus* (Blattodea: Hodotermitidae) and Its Role as A Bioindicator of Heavy Metal Accumulation Risks in Saudi Arabia. Insects. 2019; 10(2):51. 10.3390/insects10020051. 30744024 PMC6409844

[55] Marshall E. Will DNA bar codes breathe life into classification? Science. 2005; 307: 1037. 15718446 10.1126/science.307.5712.1037

[56] Bucklin A, Wiebe PH, Smolenack SB, Copley NJ, Beaudet JG, Bonner KG, et al. DNA barcodes for species identification of euphausiids (Euphausiacea, Crustacea). Journal of Plankton Research. 2007; 29(6): 483–493.

[57] Pfenninger M, Schwenk K. Cryptic animal species are homogeneously distributed among taxa and biogeographical regions. BMC Evolionary Biology. 2007; 7(1): 121. doi: 10.1186/1471-2148-7-121 17640383 PMC1939701

[58] Hebert PD, Cywinska A, Ball SL, deWaard JR. Biological identifications through DNA barcodes. Proceedings of the Royal Society B: Biological Sciences. 2003; 270(1512): 313–21. doi: 10.1098/rspb.2002.2218 12614582 PMC1691236

[59] Konecka E, Szymkowiak P. *Wolbachia supergroup* A in *Enoplognatha latimana* (Araneae: Theridiidae) in Poland as an example of possible horizontal transfer of bacteria. Scientific Reports. 2024;14: 7486. 10.1038/s41598-024-57701-y. 38553514 PMC10980700

[60] Lefoulon E, Clark T, Guerrero R, Cañizales I, Cardenas-Callirgos JM, Junker K, et al. Diminutive, degraded but dissimilar: *Wolbachia* genomes from filarial nematodes do not conform to a single paradigm. Microbial Genomics. 2020; 6(12): mgen000487. doi: 10.1099/mgen.0.000487 33295865 PMC8116671

[61] Konecka E, Ziemowit O. *Wolbachia* supergroup E found in *Hypochthonius rufulus* (Acari: Oribatida) in Poland. Infection Genetics and Evolution. 2021; 91: 104829, 10.1016/j.meegid.2021.104829. 33794350

[62] Rodrigues J, Lefoulon E, Gavotte L, Perillat-Sanguinet M, Makepeace B, Martin C, et al. *Wolbachia* springseternal: symbiosis in Collembola is associated with host ecology. Royal Society Open Science 2023; 10: 230288. 10.1098/rsos.230288. 37266040 PMC10230187

[63] Zimmermann BL, Cardoso GM, Bouchon D. et al. Supergroup F *Wolbachia* in terrestrial isopods: Horizontal transmission from termites? Evolutionary Ecology. 2021; 35: 165–182. 10.1007/s10682-021-10101-4. 33500597 PMC7819146

[64] Monika M, Bartosz N, Milena R, Uroš K, Piotr M, Tom P, et al. Taxonomic classification of the bacterial endosymbiont *Wolbachia* based on next*-*generation sequencing: is there molecular evidence for its presence in tardigrades?. Genome. 2021; 64(10): 951–958. 10.1139/gen-2020-0036. 34015229

[65] Mioduchowska M, Konecka E, Gołdyn B, Pinceel T, Brendonck L, Lukić D, et al. Playing Peekaboo with a Master Manipulator: Metagenetic Detection and Phylogenetic Analysis of *Wolbachia* Supergroups in Freshwater Invertebrates. International Journal of Molecular Sciences. 2023; 24(11): 9400. 10.3390/ijms24119400. 37298356 PMC10253400

[66] Huigens ME. et al. Natural interspecifc and intraspecifc horizontal transfer of parthenogenesis*-*inducing *Wolbachia* in *Trichogramma* wasps. Proceedings of the Royal Society B: Biological Sciences. 2004; 271: 509–515. 10.1098/rspb.2003.2640 PMC1691627 15129961

[67] Vavre F, Fleury F, Lepetit D, Fouillet P, Boulétreau M. Phylogenetic evidence for horizontal transmission of *Wolbachia* in host*-*parasitoid associations. Molecular Biology and Evolution. 1999; 16: 1711–1723. 10605113 10.1093/oxfordjournals.molbev.a026084

[68] Bleidorn C, Gerth M. A critical re-evaluation of multilocus sequence typing (MLST) eforts in *Wolbachia*. FEMS Microbiology Ecology. 2006; 10.1093/femsec/fx163 2018. 29186405

[69] Glowska E, Dragun-Damian A, Dabert M, Gerth M. New *Wolbachia* supergroups detected in quill mites (Acari: Syringophilidae). Infection Genetics and Evolution. 2015; 30: 140–146. doi: 10.1016/j.meegid.2014.12.019 25541519

[70] Rowley SM, Raven RJ, McGraw EA. *Wolbachia* pipientis in Australian spiders. Current Microbiology. 2004; 49: 208–214. 15386106 10.1007/s00284-004-4346-z

[71] Floate KD, Kyei-Poku GK, Coghlin PC. Overview and relevance of *Wolbachia* bacteria in biocontrol research. Biocontrol Science and Technology. 2006; 16: 767–788.

[72] Ren W, Wei H, Yang Y. et al. Molecular detection and phylogenetic analyses of *Wolbachia* in natural populations of nine galling Aphid species. Scientific Reports. 2020; 10, 12025. 10.1038/s41598-020-68925-z. 32694524 PMC7374581

[73] Baldo L, Werren JH. Revisiting *Wolbachia* supergroup typing based on *WSP*: spurious lineages and discordance with MLST. Current Microbiology. 2007; 55,81–7. 17551786 10.1007/s00284-007-0055-8

[74] Werren JH, Bartos JD. Recombination in *Wolbachia*. *Current Biology*. 2001; 11: 431–435. 11301253 10.1016/s0960-9822(01)00101-4

[75] Kondo N, Tuda M, Toquenaga Y, Lan YC, Buranapanichpan S, Horng SB, et al. *Wolbachia* infections in world populations of bean beetles (Coleoptera: Chrysomelidae: Bruchinae) infesting cultivated and wild legumes. Zoological Science. 2011; 28:501–508 doi: 10.2108/zsj.28.501 21728798

[76] Kageyama D, Narita S, Imamura T, Miyanoshita A. Detection and identification of *Wolbachia* endosymbionts from laboratory stocks of stored*-*product insect pests and their parasitoids. Journal of Stored Products Research. 2010; 46:13–19. doi: 10.1016/j.jspr.2009.07.003

[77] Kondo N, Ijichi N, Shimada M, Fukatsu T. Prevailing triple infection with Wolbachia in *Callosobruchus chinensis* (Coleoptera: Bruchidae). Molecular Ecology. 2002;11:167–180 doi: 10.1046/j.0962<italic>-</italic>1083.2001.01432.x 11856419

[78] Numajiri Y, Kondo NI, Toquenaga Y. Melanic mutation causes a fitness decline in bean beetles infected by *Wolbachia*. Entomologia Experimentalis et Applicata. 2017; 164:54–65. doi: 10.1111/eea.12588

[79] Numajiri Y, Kondo NI, Toquenaga Y. et al. Intraspecies variation in cytoplasmic incompatibility intensity in the bean beetle *Callosobruchus analis*. Evolutionary Ecology. 2024; 10.1007/s10682-024-10311-6.

[80] Malloch G, Fenton B. Super infections of *Wolbachia* in byturid beetles and evidence for genetic transfer between A and B supergroups of *Wolbachia*. Molecular Ecology. 2005; 14(2): 627–637. 15660951 10.1111/j.1365-294X.2005.02432.x

[81] Bordenstein SR, Marshall ML, Fry AJ, Kim U, Wernegreen JJ. The tripartite associations between bacteriophage, *Wolbachia*, and arthropods. PLOS Biology. 2006; 2: 384–393. 10.1371/journal.ppat.0020043 PMC1463016 16710453

[82] Werren JH, Zhang W, Guo L. Evolution and phylogeny of *Wolbachia*: reproductive parasites of arthropods. Proceedings of the Royal Society of London. Series B. 1995b; 261: 55–63. 7644549 10.1098/rspb.1995.0117

[83] Dobson SL, Bourtzis K, Braig HR, Jones BF, Zhou W, Rousset F, et al. *Wolbachia* infections are distributed throughout insect somatic and germ line tissues. Insect Biochemistry and Molecular Biology. 1999; 29: 153–160. 10196738 10.1016/s0965-1748(98)00119-2

[84] Turelli M, Hoffmann AA. Cytoplasmic incompatibility in *Drosophila simulans*: dynamics and parameter estimates from natural populations. Genetics. 1995; 140: 1319–1338. 7498773 10.1093/genetics/140.4.1319 PMC1206697

[85] Hoffmann AA, Clancy DJ, Merton E. Cytoplasmic incompatibility in Australian populations of *Drosophila melanogaster*. Genetics. 1994; 136(3): 993–999. 8005448 10.1093/genetics/136.3.993 PMC1205902

[86] Fine PEM. On the dynamics of symbiote*-*dependent cytoplasmic incompatibility in Culicine mosquitoes. Journal of Invertebrate Pathology. 1978; 30: 10–18. 10.1016/0022-2011(78)90102-7 415090

[87] Rasool B, Qayyum I, Iqbal J, Rasool I. Impact of diets and environmental variables on the biology of *Callosobruchus maculatus* (F). (Coleoptera: Chrysomelidae). International Journal of Pest Management. 2024; 1–11. doi: 10.1080/09670874.2024.2334240

[88] Misailidis M, Kotsiou N, Moulistanos A, Gewehr S, Augustinos AA, Mourelatos S, et al. The Molecular Detection, Characterization, and Temperature Dependence of *Wolbachia* Infections in Field Populations of *Aedes albopictus* (Diptera: Culicidae) Mosquitoes in Greece. Diversity. 2024; 16(1): 43. 10.3390/d16010043.

[89] Kristensen TN, Loeschcke V, Tan Q, Pertoldi C, Mengel-From J. Sex and Age Specific Reduction in Stress Resistance and Mitochondrial DNA Copy Number in *Drosophila melanogaster*. Scientific Reports. 2019; 9: 12305. 31444377 10.1038/s41598-019-48752-7 PMC6707197

[90] Li ZH, Zhang P, Ma HK, Xu WY, Sun JQ, Yan BL, et al. Effect of Temperature and Salinity on MtDNA Copy Number of the Ridgetail White Prawn, *Palaemon carinicauda* Holthuis, 1950 (Decapoda, Palaemonidae). Crustaceana. 2018; 91: 1061–1072.

[91] Syromyatnikov MY, Gureev AP, Mikhaylov EV, Parshin PA, Popov VN. Pesticides Effect on the Level of MtDNA Damage in Bumblebees Heads (*Bombus terrestris* L.). Periodico Tche Quimica. 2020; 17: 395–402.

[92] Morrow JL, Frommer M, Royer JE, Shearman DC, Riegler M. *Wolbachia* pseudogenes and low prevalence infections in tropical but not temperate Australian tephritid fruit flies: manifestations of lateral gene transfer and endosymbiont spillover? BMC Evolutionary Biology. 2015; 15(1): p.1. 26385192 10.1186/s12862-015-0474-2 PMC4575488

